# Evaluation of the protective efficacy of a transfluthrin-based spatial repellent product to reduce malaria prevalence in Uganda: study protocol for a cluster-randomised double-blinded control trial—the Mossie-GO trial

**DOI:** 10.1186/s13063-025-09365-w

**Published:** 2026-02-03

**Authors:** Jessica Dennehy, Will Dyall, Akin Jenkins, John Bradley, Asadu Sserwanga, Ruth Kigozi, John Baptist Bwanika, Anthony Nuwa, Henry Mawejje, Matthew A. Turner, Richard Wallace, Frederick G. A. Lyle, Alexandra Hiscox, Alastair K. Livesey, James G. Logan, Jane Achan, Robert T. Jones

**Affiliations:** 1 Arctech Innovation Ltd, London, UK; 2 London School of Hygiene & Tropical Medicine, London, UK; 3 Malaria Consortium, Kampala, Uganda; 4 Loughborough University, Leicestershire, UK; 5 KuaSolar Ltd, London, UK; 6 Africa Power Ltd, Crawley, UK

**Keywords:** Malaria, Spatial Repellent, Transfluthrin, Vector Control, Uganda, Solar, RCT

## Abstract

**Background:**

Progress towards elimination and eventual eradication of malaria is threatened by challenges such as the rise in insecticide resistance and low coverage of existing vector control tools. Spatial repellents offer personal and household protection against biting mosquitoes by disseminating repellents into a given area. The trial described here aims to evaluate the efficacy of an active transfluthrin-based spatial repellent device (Mossie-GO™) against malaria in Uganda, using a placebo-controlled, double-blinded cluster randomised control trial. The study’s primary objective is to demonstrate and quantify the protective efficacy of Mossie-GO™ in reducing the prevalence of malaria infection in children ≤ 5 years of age. The study’s secondary objectives are to measure the impact of the intervention on entomological correlates of transmission, to determine user acceptance of the device and to quantify transfluthrin concentration in the air.

**Methods:**

The trial has fifty-six clusters randomly assigned in a 1:1 ratio to either the intervention or placebo-control arm. One hundred children at baseline and sixty children ≤ 5 years of age will be sampled in each cluster at 6 and 12 months to measure the primary endpoint. Each child will be sampled from a different household to avoid within-house replication. A subset of households from each cluster will be selected for secondary endpoint sampling. All households enrolled into the study will be encouraged to continue use of other malaria control tools.

**Discussion:**

Trial results will contribute to the growing research on spatial repellent efficacy in sub-Saharan Africa and will inform recommendations for the use of spatial repellents in malaria control, specific to rural and peri-urban contexts in Uganda. Information on household characteristics, behaviour related to malaria exposure and user acceptability of the intervention will also be collected to improve understanding of the intervention usage and impact. Following the trial, results will be publicly disseminated.

**Trial registration:**

The trial is registered with ClinicalTrials.gov 01/04/2024 unique identification (ID): NCT06232954.

## Administrative information

Note: the numbers in curly brackets in this protocol refer to SPIRIT checklist item numbers. The order of the items has been modified to group similar items (see http://www.equator-network.org/reporting-guidelines/spirit-2013-statement-defining-standard-protocol-items-for-clinical-trials/).

**Table:** 

Title {1}	Evaluation of the protective efficacy of a spatial repellent to reduce malaria prevalence in Uganda: Study protocol for a cluster-randomized double-blinded control trial: The Mossie-GO Trial
Trial registration {2a and 2b}.	The trial is registered with ClinicalTrials.gov 01/04/2024 unique ID: NCT06232954.
Protocol version {3}	Version 2.0, 06th February 2025
Funding {4}	This study is funded by an Innovate UK grant awarded to Africa Power and Arctech Innovation (Project Title: Trials of Solar-Powered, Active, Spatial Mosquito Control Dispensers to Reduce Malaria, Application Number: 10053619). Innovate UK is the UK’s innovation agency which helps companies to grow through their development and commercialisation of new products.
Author details {5a}	JD, RTJ, AH, AJ, WD, JGL: Arctech Innovation Ltd, London, United Kingdom JA, AS, JBB, RK, HM, AN: Malaria Consortium, Kampala, Uganda RTJ, JGL, JB: London School of Hygiene & Tropical Medicine, London, United Kingdom AKL: Africa Power Ltd, Crawley, United Kingdom RW, FGAL: KuaSolar Ltd, London, United Kigndom MAT: Loughborough University, Leicestershire, United Kingdom
Name and contact information for the trial sponsor {5b}	Africa Power Ltd, Contact name: Alastair Livesey Address: Griffin House, 1035 High St, Crawley RH10 1DQ Tel: + 44 (0) 1403 864,702 Email: alivesey@africapowerltd.com
Role of sponsor {5c}	Africa Power, as the study sponsor had no involvement in developing the study design; collection, management, analysis, and interpretation of data; writing of the report; and the decision to submit the report for publication.

## Introduction

### Background and rationale {6a}

Malaria remains one of the greatest healthcare problems facing the African continent, with over 260 million people contracting the disease annually [1]. In 2023 alone, 597,000 deaths were attributed to malaria with 94% of these infections and 95% of deaths occurring in the World Health Organization (WHO) African region [1]. Sub-Saharan Africa (SSA) bears the highest burden of morbidity and mortality worldwide, despite improvements with diagnostics, treatment, vaccines and large-scale deployment of vector control measures. Children and pregnant women remain the highest risk groups, with children under 5 accounting for about 80% of all malaria deaths in the African region. The burden of malaria also greatly impacts gross domestic product and economic growth. It reduces productivity by causing illness and death, increasing healthcare costs and hindering education [2, 3].

Uganda has one of the world’s highest malaria incidence rates of 478 cases per 1000 population per year [4]. It is estimated that there were approximately 12,573,000 malaria cases in Uganda in 2023, which accounted for 4.8 of global malaria cases [5]. The entire population is considered to be at risk of contracting malaria. The primary malaria vectors in Uganda are *Anopheles gambiae s.s.*, *Anopheles arabiensis* and *Anopheles funestus s.l*. [6]. Progress towards elimination and eventual eradication of the disease is threatened by challenges such as the rise in insecticide resistance and low coverage of existing vector control tools [7]. New vector control tools are, therefore, urgently needed to address the growing threat of insecticide resistance and target outdoor biting vectors.

Spatial repellents offer a promising solution to complement existing vector control tools in contexts where widely used vector control strategies, such as insecticide-treated nets (ITNs) and indoor residual spraying (IRS) are becoming less effective. Spatial repellents are intended to be used in combination with existing WHO-recommended vector control methods and the WHO has recently recommended methods to evaluate the efficacy of new spatial repellent products. Spatial repellents reduce human-vector contact by repelling the vector from the chemical stimuli created by a host, interfering with host detection, inhibiting attraction and/or reducing feeding response [8, 9]. This mode of action is expected to provide protection against daytime and early evening biting vectors as well as protection in enclosed/semi enclosed and peri-domestic spaces. Several active ingredients have been shown to provide spatial repellent effects and to provide protection from mosquito bites; for example, the use of transfluthrin-based passive emanators has led to reduced biting in a wild pyrethroid-resistant strain of *Anopheles arabiensis* mosquitoes in semi-field trials in Tanzania and field trials in Benin [10, 11] Additionally, the use of a metofluthrin-based spatial repellent almost completely prevented *Anopheles funestus* landing indoors and ten times lower landing rates within 10 m of the emanator outdoors [12]. These findings suggest that transfluthrin-based control interventions may be effective even in areas of pyrethroid resistance. Preliminary findings have also suggested efficacy against vectors of both malaria and *Aedes*-borne viruses [13–15].

Noted limitations with spatial repellents include the limited residual effect of the repellent and the need for external power or heat for diffusion of the volatile active ingredients if they are to be actively dispersed, which may be required particularly at cooler temperatures [16]. However, nearly 70% of households in SSA are off-grid [17]. The intervention proposed in this study (Mossie-GO™) allows for active emanation of transfluthrin into the air, powered by off-grid solar power technology. Although similar types of devices are widely available in Europe, USA, Australia and many other countries, their availability in low-middle-income countries (LMICs) is lower due to a perceived lack of market and energy requirements. The main benefit of solar energy is that the devices can be charged throughout the day and then, once turned on, will release the active compound and be used to provide protection to members of households at times of day when they are not under a bed net. A further advantage is the lack of installation required; the solar panel that accompanies Mossie-GO™ is portable and can be placed outside by the user, rather than being fitted to a fixed location. The solar panel can then be easily connected to the portable Mossie-GO™ device for charging. A powered device is expected to provide greater protection than a passive device because the fan enables a controlled and continuous distribution of the active ingredient. This study will serve as a proof-of-principle of the efficacy of an active transfluthrin-based spatial repellent device (Mossie-GO™) against malaria in Uganda.

### Objectives {7}

The study’s primary objective is to demonstrate and quantify the protective efficacy of Mossie-GO™, a transfluthrin-based active spatial repellent device, in reducing prevalence of malaria infection in children ≤ 5 years of age, as determined by rapid diagnostic test (RDT) and microscopy. Secondary objectives are incorporated to measure the impact of the intervention on entomological correlates of transmission, user acceptance of the device and quantification of transfluthrin concentration in the air. Secondary objectives are:

1. To compare mosquito species composition, population density and sporozoite rates in mosquitoes caught using indoor CDC-light traps and Human Landing Catches (HLC) between intervention and control arms.
2. To measure the prevalence of resistance to pyrethroids in *Anopheles* mosquitoes using WHO tube test bioassay.
3. To collect information on subject behaviour related to exposure to malaria and use of existing control tools via household surveys.
4. To quantify temporal changes in concentration of transfluthrin present in the air over an evening and residual concentrations over the duration of the study and measure the correlation between indoor temperature and the concentration of active in the air.
5. To assess the acceptability of Mossie-GO™ in the study population as an indicator of whether this device could be scaled up in similar settings in the future using surveys, interviews and focus group discussions (FGDs).
6. To assess the safety of intervention through monitoring of adverse events (AEs) and serious adverse events (SAEs).

### Trial design {8}

**Fig. 1:**
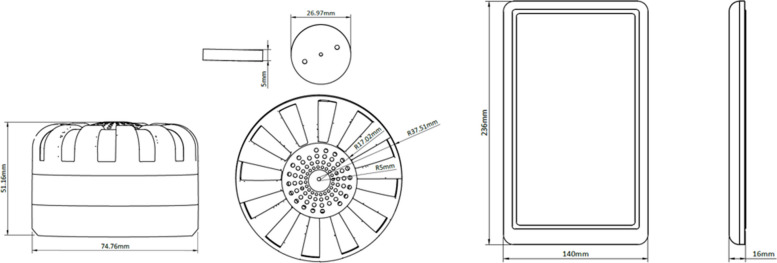
Diagram of the Mossie-GO™ device (left), containing a fan and transfluthrin-treated or control disc, powered by a solar panel (right)

This trial is a double-blinded placebo controlled cRCT with two arms. A total of 56 clusters will be randomly assigned, in a 1:1 ratio, to one of the two arms to receive the intervention or the placebo control. The intervention is a Mossie-GO™ device, containing a transfluthrin-treated disc which sits above a fan powered by solar energy (Fig. 1). The control arm will receive the Mossie-GO™ device with an identical looking disc with no active ingredient. All eligible, consenting households will receive a single Mossie-GO™ device with 5 W solar panel, and discs will be replaced at monthly intervals. The device requires 2 h charging time during the day and should be switched on in the evening and placed in the room where the child ≤ 5 years of age sleeps. If multiple children ≤ 5 years of age sleep in different locations, one location should be selected. If there are no children ≤ 5 years of age within the household, the Mossie-GO™ device can be placed in an alternative sleeping room.

A survey will be conducted where 100 children ≤ 5 years of age in each cluster will be tested for malaria using RDTs and microscopy to determine malaria prevalence at baseline. Sampling will be repeated for 60 children ≤ 5 years of age in each cluster at 6 and 12 months. A household survey, classifying housing structure and collecting other variables which may impact the efficacy of the intervention, will be conducted within the sampled households. At the same timepoints, indoor CDC light traps will be installed in 7 households selected at random in each cluster and run from 7 pm to 7 am to collect mosquitoes. HLCs will be conducted over 3 consecutive nights indoors and outdoors of 3 households selected at random in 10 clusters. Households will only be eligible for HLC sampling if they have been selected for CDC sampling and sampling will be conducted on separate nights. Air sampling will also be conducted within the households selected for HLC sampling at months 9 and 12 to quantify the concentrations of transfluthrin in the air and to help inform entomological and epidemiological outcomes. Acceptability FGDs and surveys to understand barriers to use of the Mossie-GO™ device will also be conducted at months 6 and 12.

## Methods: participants, interventions, and outcomes

### Study setting {9}

The study will be conducted in Uganda across two malaria transmission seasons in the districts of Jinja and Buikwe. Malaria is the leading cause of sickness and death in Uganda and is responsible for up to 40% of all outpatient visits, 25% of hospital admissions and 14% of all hospital deaths. The malaria death rate in Uganda is estimated to be between 70,000 and 100,000 deaths per year [4]. The entire population is considered to be at risk of contracting malaria [18].

There is stable perennial malaria transmission in 90–95% of the country, and low and unstable transmission with potential for epidemics in the remaining 5–10% of the country. The transmission peaks are aligned with the two annual rainy seasons in March–May and September–November [18].

The population of the study areas identified in Uganda for this study has a mixture of urban, peri-urban and rural communities. Household structures vary in size, wall and ceiling material, room number and window structures. Community members also vary in occupation, ethnic group and utilisation of routine malaria control interventions.

There was community engagement in the development of the protocol, with Village Health Team members contributing to selection of clusters, selection of blood sampling locations within clusters and the wording to be used in data collection forms. User feedback was also important in development of the Mossie-GO™ device, and was obtained in Songea, Tanzania.

### Eligibility criteria {10}

To be eligible for enrolment, participants needed to meet inclusion criteria of age (0 months to ≤ 5 years), no plans for extended travel (> 1 month) outside of home during the study, not participating in another clinical trial or being on regular malaria prophylaxis, and parents or guardians providing written consent.

### Who will take informed consent? {26a}

Informed consent will be obtained by members of the VHT and data collectors from the local districts. This existing framework will be valuable for community sensitisation and ongoing study implementation. Teams of data collectors will be supervised by local research assistants, who will report to the technical officer. Following national, district and village level sensitisation, all households within the community will be presented with a Participant Information Sheet (PIS) and Informed Consent Form (ICF) containing information on the general purpose and methods of the study, translated into the local language so that it may be read by participants or read aloud to them if preferred. The head of household will be asked to provide their written informed consent for their household to participate in the study. Providing consent will enable participants to receive the Mossie-GO™ device, the subsequent visits for disc refill and to participate in the household and acceptability survey. Once enrolled, the household will receive a household ID number. Once enrolled into the study, written informed consent will be obtained separately for all other study activities using separate PIS and ICFs. In total, up to six types of consent may be given:

1. Head of household to provide household consent for use of device and participation in the household and acceptability survey (compulsory to take part in the study).
2. Parent/caregiver/guardian of child to provide consent for malaria prevalence sampling at 0, 6 and 12 months via RDT, microscopy and polymerase chain reaction (PCR) (maximum 5600 households, made up of 100 households at baseline and 60 households at subsequent timepoints per cluster with a child ≤ 5 years of age at enrolment in the study, randomly selected at each of the sampling points).
3. Head of household to provide consent for mosquito collection via CDC light trapping (392 households, made up of 7 households in 56 clusters, randomly selected with replacement at each of the sampling points).
4. Head of household to provide consent for mosquito collection via HLCs (30 households, made up of 3 houses in 10 clusters, randomly selected from the subset of households undergoing CDC light trapping with replacement at each of the sampling points).
5. Head of household to provide consent for air sampling (28 households, made up of 2 households in 14 clusters, randomly selected from a subset of the households undergoing HLCs with replacement at each of the sampling points).
6. Head of household, or caregivers or other community members to provide consent for FGDs to assess acceptability of the intervention (8–12 FGDs will take place containing 6–10 participants).

Parent/guardian of a participant may refuse to answer any question or withdraw from the study at any time without any impact to the participant. Study staff can terminate subject participation at any time during the study period if a participant no longer meets the inclusion criteria and/or based on adverse event (AE) or serious adverse event (SAE) assessment.

### Additional consent provisions for collection and use of participant data and biological specimens {26b}

No additional consent will be sought for use of participant data and biological specimens. The PIS will inform the participants that blood samples will be tested in the laboratory in Uganda and destroyed after analysis is completed. The records concerning household/child participation are to be used only for the purpose of this research project. Names will not be used on labels on samples or laboratory specimens or in any report resulting from this study. Only authorised members of the study team will have access to information linking the child’s name with the participants’ identification number. After completion of the collection of material, data will be anonymised and identifying information will be destroyed. Other material and data from this study will then be kept for 10 years. This is a legal requirement of the funder. All data will be collected, processed, stored and handled in accordance with the Data Protection Act 2018.

## Interventions

### Explanation for the choice of comparators {6b}

This trial will use a placebo control to reduce the impact of behavioural factors on trial outcomes. The control arm will still receive a Mossie-GO™ device, but the discs will contain no active ingredients. The device containing the untreated disc is not expected to prevent mosquito bites. A placebo device was selected over no control device to mitigate against altered behaviour in the control arm, to allow for better measure of intervention impact. Participants will be informed that half of recruited households will receive a placebo, and all participants will also be encouraged to continue use of standard care to ensure control group participants are not put at any additional risk.

### Intervention description {11a}

The Mossie-GO™ repellent device is approximately 75 mm × 51 mm and can be fitted with repellent discs impregnated with transfluthrin and a carrier oil. These discs sit above a fan that is powered by a small motor charged by solar energy. The device, containing treated discs, is expected to both prevent bites and cause some mortality to mosquitoes when switched on for 8–12 h overnight. The trial design includes a placebo product of matched design to the Mossie-GO™ device but with discs containing no active ingredient or carrier oil.

At baseline (month 0) both the intervention and control arms will receive the Mossie-GO™ and a disc to last 1 month. Discs will be replaced on a monthly basis and old discs will be discarded by the study team.

### Criteria for discontinuing or modifying allocated interventions {11b}

Any parent or guardian of a participant may refuse to answer any question on the ICFs or study surveys or withdraw from the study at any time without any impact to the participant. Study staff can terminate subject participation at any time during the study period if a participant no longer meets the inclusion criteria and/or based on AE/SAE assessment.

Since this is a cross-sectional study, if a participant withdraws or their participation is terminated at any time point, they can be replaced to ensure the desired sample size is met. If a participant withdraws, their data will still be used in the final analysis, unless the participant’s parent/guardian requests for the data to be removed from the study. In this case, an additional participant can be recruited, following the protocol to obtain informed consent, to meet the desired sample size. Participant data will still be stored, anonymised, for 10 years. Parents/guardians of withdrawn participants can request for data to be removed sooner, at their discretion. Withdrawn participants will be followed up for the purpose of adverse event monitoring only. As payments are being made to participants in instalments of 5000 Ugandan shillings (UGX; ~ 1.36 USD) at the time of each cross-sectional survey over the four planned surveys, participants will still receive payment for the relevant participation even if they withdraw at a later time point in the study. Participants will not be paid in advance in the case they withdraw before sampling has been conducted.

### Strategies to improve adherence to interventions {11c}

Study team and VHT members will verbally explain the use of the Mossie-GO™ device in the local language, and a user manual covering the solar panel and the Mossie-GO™ device, including images, will be provided to households to inform of its use. Participants will be given the opportunity to ask questions at the time of enrolment and provided with study team contact phone numbers during the informed consent process. Participants will receive monthly visits from VHTs to monitor AEs which will provide an opportunity to check the use of the device or for participants to report problems. At the 6-month time point, an acceptability survey will be conducted to identify any barriers to adherence and provide an opportunity to amend the protocol to reduce barriers.

### Relevant concomitant care permitted or prohibited during the trial {11d}

The study design will not withhold standard-of-care for clinical management of malaria or vector control interventions (ITNs, IRS or larviciding) in either control or intervention arms; however, use of other interventions will be monitored and recorded throughout the study period. Study participants will also be encouraged to adhere to standard vector control interventions (such as use of ITNs). This will allow for evaluation of the impact of the spatial repellent device assuming all other measures are still occurring for malaria prevention, providing insight into the additive benefit above that provided by currently recommended WHO malaria preventative measures and further exploring the impact of using multiple interventions.

### Provisions for post-trial care {30}

This is not applicable—the study will not provide post-trial care.

### Outcomes {12}

The primary outcome measured in this study will be the average reduction in malaria infection prevalence in children ≤ 5 years of age from baseline to month 12, as measured by RDT and microscopy. Secondary outcome measures include:

1. CDC light trap indoor density for anophelines and by anopheline species, as measured by light trap collections from 7 pm to 7 am on a 6 monthly basis. Change in mosquito density at baseline and month 12 between arms will be analysed by negative binomial regression with a random effect for study cluster.
2. Anopheline-human contact (indoor and outdoor) using human biting rate (HBR) as an indicator for all anophelines and by anopheline species, as measured by HLC during 6-h intervals on a 6-monthly basis. Change in HBR at baseline and month 12 between arms will be analysed by negative binomial regression with a random effect for study cluster.
3. Anopheline infectivity using sporozoite rate as an indicator for all anopheline and by anopheline species, as measured by laboratory detection of sporozoites in mosquito heads and thoraces from a subsample of anophelines collected during CDC light trap and HLC collections.
4. Insecticide resistance, as measured by WHO tube test bioassay, during the intervention phase. Confirmed resistance, possible resistance and susceptibility will be calculated through evaluating knockdown and mortality rates.
5. Subject behaviour and household features related to exposure to malaria and use of existing control tools, as measured by quantitative and qualitative surveys on a 6-monthly basis. Survey outputs will be summarised using basic statistics.
6. Concentration of transfluthrin in air over the duration of an evening’s use or the Mossie-GO™ and the impact of indoor temperature on the concentration, as measured by air sampling at months 9 and 12. Mean concentration will be compared between arms.
7. Acceptability of Mossie-GO™ in these communities, as measured be quantitative and qualitative surveys at 6 and 12 months and FGDs at 12 months. Outputs will be summarised using basic statistics and thematic analysis.
8. Safety of intervention through monitoring of AEs and SAEs. Measured actively and passively throughout the duration of the study and total number of events and type of events will be compared between study arms.

### Participant timeline {13}

**Table:** 

Month	Study period				
	Enrolment/Baseline data collection	Intervention			Close out
	0 (June 2024)	6 (Dec 2024)	9 (March 2025)	12 (June 2025)	July–Oct 2025
*Enrolment:*					
Household enrolment	X				
Device distribution	X				
U5 eligibility screen	X				
U5 enrolment	X				
Baseline U5 blood sampling	X				
Household survey	X				
CDC light trapping enrolment	X				
Baseline CDC light trapping	X				
HLC enrolment	X				
Baseline HLCs	X				
Air sampling enrolment			X		
*Intervention:*					
Blood sampling		X		X	
Household survey		X		X	
CDC light trapping		X		X	
HLCs		X		X	
Insecticide resistance sampling				X	
Air sampling			X	X	
Acceptability survey		X		X	
FGDs				X	
*Assessments:*					
Final analysis					X
Dissemination of results within community					X

## Sample size {14}

Data collected from the study region suggest prevalence of malaria infection by RDT in children ≤ 5 years of age is 20%. A conservative coefficient of variation (CV) of 0.5 for between cluster prevalence was assumed. To detect a 40% reduction in prevalence with 90% power, we need 28 clusters per arm and a sample of 100 children in each cluster. Due to budget restrictions, sample size was reduced after baseline to 60 children in each cluster at 6 and 12 months, resulting in 86% power.

Entomological sample sizes were determined based on logistics, considering budget and personnel restrictions. The sample sizes of similar studies were also considered. For CDC light trapping, sampling will be carried out in all clusters and randomised at the household level. HLCs will be randomised at both the cluster and household level.

### Recruitment {15}

Community sensitisation will take place by the study team and VHTs communicating the study purpose and activities and will also involve local leaders. This will be done at multiple levels including centrally at the Ministry of health, district level and the village level. Active recruitment will take place through the provision of information by designated study staff to central points of the community, such as community leaders, health workers, schools and churches. Information will then be further provided to parent(s)/guardian(s) of potential participants through word of mouth to provide potential study participants further information on the study. Clusters have been identified where there are more than 150 houses with a child ≤ 5 years of age, to ensure that the sample size will still be met in the case a proportion of households do not consent to primary endpoint sampling.

## Assignment of interventions: allocation

### Sequence generation {16a}

All randomisation procedures will take place electronically. Treatment and control will be assigned as blinded, variables A and B, by the trial data manager and details of this assignment will be recorded in a sealed envelope and will not be shared with or accessible to any other team members.

The unit of randomisation is a cluster, corresponding approximately to villages within the study area. Clusters will be randomly assigned to two study arms, control and treatment intervention, using a computer-assisted constrained randomisation approach, facilitated by R [19]. To ensure adequate and feasible sampling, cluster selection will be restricted based on population size and population stability (Appendix Table 1). To reduce the impact of confounding factors, clusters will be stratified by land type (urban or rural) and study district, ensuring equal representation across both study arms. No significant inter-cluster variation was identified, meaning household selection within clusters will proceed as simple random sampling without blocking. This randomisation procedure involves generating 1000 unique sets of random treatment assignments. In each cycle, the differences in treatments allocated between district and land type strata will be assessed and the iteration with the least disparity, considered the most well-balanced iteration, will be selected as the final scheme for assigning treatments. Additional confounding factors will be factored into the statistical analysis (Appendix Table 2).

### Concealment mechanism {16b}

Investigators, study staff, statistician and study participants will be blinded to treatment allocation of clusters. The intervention and control will be deployed in houses by blinded study personnel using a blinded coding system. Following completion of the trial and locking of the database, the study statistician will conduct an unblinded analysis.

### Implementation {16c}

The distribution of the intervention will occur immediately following baseline data collection, and thus verification of underlying assumptions of study power (prevalence and CV) will not be verified prior to distribution. Analysis of baseline data will take place prior to the first round of intervention data collection, providing an opportunity to revise sample sizes if necessary. Within a cluster, all consented households with a child ≤ 5 years of age will receive a device regardless of whether they are randomly selected for sampling at the baseline time point. Where spare Mossie-GO™ devices are available, households without a child ≤ 5 years of age will also be given a device. As per the manufacturer’s guidance, the device requires 2 h charging time during the day and should be switched on in the evening and placed in the room with the child ≤ 5 years of age. If multiple children ≤ 5 years of age sleep in different locations, one location should be selected. If there are no children ≤ 5 years of age within the household, the Mossie-GO™ device can be placed in an alternative sleeping room. Trained VHTs and data collectors will be responsible for deployment of the product and monthly refill/disposal of the discs at monthly intervals within the sample populations to ensure adherence. Reviews of device use will be conducted to inform any retraining requirements.

## Assignment of interventions: blinding

### Who will be blinded {17a}

This is a double-blinded trial in which the trial participants and all study staff will be blinded, with the exception of two delegated unblinded study staff members. These include the two members of staff responsible for coordinating the manufacture and distribution of the Mossie-GO™ to either the treatment or control cluster. The data manager who implements the allocation sequence and the statistician responsible for analysis will remain blinded until the completion of data collection and the locking of the database at the end of the study.

### Procedure for unblinding if needed {17b}

Study unblinding will occur in the case of an AE or SAE, at the discretion of the principal investigators (PIs). The site clinician will review all AEs and SAEs and inform the PI of the AE/SAE under consideration within 24 h. If deemed necessary, a delegated unblinded study staff member will determine if the participant and household allocation are within the control or intervention arm. If they are within the intervention arm and deemed necessary by the PI and site clinician after considering the type and frequency of event and reviewing the likelihood of the adverse event to be related to the study, action will be taken such as study termination or device withdrawal until further investigation. All cases of study unblinding will be well documented using an unblinding log and considered within the study analysis, for example, in the case a whole cluster becomes unblinded through participant communication. Documentation of the unblinding will be reported to the data safety monitoring board (DSMB) and international research board (IRB).

## Data collection and management

### Plans for assessment and collection of outcomes {18a}

#### Description of clusters prior to baseline data collection

Prior to enrolment and baseline data collection, a total of 56 clusters will be identified using pre-trial census data, to ensure there are enough households with children ≤ 5 years of age to meet the sample population. Additional household enumeration activities will be conducted to validate this information, and clusters will be reselected where necessary. Clusters will be defined as discrete villages and mapped using mapping software (ArcGIS/QGIS) [20, 21]. Households will be enrolled to the trial by obtaining consent from the head of household, which will enable the household to receive the Mossie-GO™ device. Once enrolled into the trial, households will receive a unique identification number which will be used for all future data collection activities.

#### Malaria prevalence monitoring

One hundred children per cluster at baseline and sixty children per cluster at months 6 and 12, selected at random from separate households, will be sampled for malaria infection. A master list of households will be generated listing households with children ≤ 5 years of age and households will be randomly selected with replacement at each of the time points. If there are multiple children ≤ 5 years of age within the household, the Kish Grid method will be used to select the participant [22]. The study will be explained and consent obtained from the child’s parent/guardian before enrolling the child in the study. Once enrolled, the age and sex of the participant, along with use of other vector control products and antimalarial drugs will be recorded. A fingerstick blood sample will be taken for malaria RDT, a blood smear for analysis by microscopy and a blood spot for analysis by PCR at a later date. All participants will be eligible for malaria treatment, if they test positive by RDT at any sampling time point. This will be accessed following national standard of care guidance at their local health care facility or through the VHTs. Trained laboratory technicians will be responsible for blood sampling, reading and recording results and sample analysis via microscopy and PCR. Microscopic examination of slides will be conducted two times, independently in verified reference laboratories. Participant data and results will be collected manually on paper logs and then entered into Open Data Kit (ODK) software [23].

#### Household survey

A questionnaire will be presented to the head of household at the point of malaria sampling to assess household characteristics and behaviours which may impact study outcomes. Questions within the survey will include, but not be limited to housing classification (urban, peri-urban, rural), housing characteristics (size, ventilation, construction materials), family structure (multiple families inhabiting one household, one family inhabiting multiple household structures etc.), number of children ≤ 5 years of age sleeping in the room with the Mossie-™unit, inhabitant(s) occupation(s), use of alternative vector control tools, whether occupants slept under a bed net the night before the survey, socio-economic status, number of inhabitants and their age and presence of domesticated animals (density and location). This survey will be conducted by the data collectors responsible for obtaining informed consent. All data will be inputted directly to forms on ODK.

#### Acceptability of Mossie-GO™

##### Surveys/interviews

A questionnaire will be presented to the head of household at the point of malaria sampling at months 6 and 12 to assess the household’s experience and opinions of the Mossie-GO™ device. This will explore whether households have had positive or negative experiences with Mossie-GO™, whether household members would recommend Mossie-GO™ to other people and willingness to pay. This survey will be conducted by the data collectors responsible for obtaining informed. All data will be entered directly to forms on ODK.

##### Focus group discussions

FGDs will also be conducted to explore acceptability of the intervention, including factors such a willingness to pay and product design. Approximately 8–12 FGDs will take place, containing 6–10 participants. Groups will include a range of participants, for example, local community members, VHTs and district health officials. The discussions will be audio-recorded and transcribed.

##### Entomological monitoring

Within each of the 56 clusters, CDC light trap collections will be conducted in 7 households from 7 pm–7 am at baseline, 6 and 12 months to allow assessment of the impact of intervention on the density of *Anopheles* mosquitoes indoors. Data collectors will be trained in setting up and taking down CDC light traps and heads of households may be trained to switch on/off the traps at the required time when necessary. Households will be selected at random for CDC light trapping at each timepoint. Additionally, HLCs will be performed indoors and outdoors for 3 consecutive nights in 3 households within each of 10 clusters (30 households total) at baseline, 6 and 12 months to determine the effect of the intervention on the host seeking behaviour of mosquitoes. Alongside the data collector, the head of household and community members will be trained to conduct the HLC and will be supervised during data collection. The clusters and households selected for HLCs will be selected at random for each time point.

Caught mosquitoes from CDC light trapping and HLCs will be examined to record vector species diversity, density and sporozoite rates at each time point. To characterize the insecticide resistance profile of the vector population in the study area, a series of WHO tube tests will be conducted in both districts (Jinja and Buikwe). Larval dipping will be conducted at selected sites, with larvae reared to adults in laboratory facilities and tested for insecticide resistance following WHO protocols and using standard WHO data collection forms [24].

##### Air sampling

A geo-spatial temporal profile of transfluthrin concentrations measured in nanograms of diffused transfluthrin will be generated at the 9- and 12-month time points. Air sampling will be conducted in a total of 28 households, made up of 2 households from 14 clusters, selected from the same pool of households undergoing HLCs. Sampling will be conducted at two time intervals, 1 h after device start and then automatically 4 h after that, to determine variation in air availability of transfluthrin. Sampling will take place at a distance between 1 and 4 m from the Mossie-GO™ device and 0.5 m above the ground. After collection, samples will be stored at − 20 °C.

To determine wind direction and strength of air movements, as well as temperature during collections, a long-range wireless wind logger will be set up at 1–4 m height next to the device during the air sampling period. Logging of parameters will be done in 5-min intervals.

### Plans to promote participant retention and complete follow-up {18b}

Participants will receive compensation on completion of each sampling period, i.e. baseline, 6 and 12 months. This will promote participant retention and participation in the next sampling period. They will also be visited monthly by their local VHTs, which will provide an opportunity to raise any concerns or ask questions.

### Data management {19}

#### Data entry

Data will be captured electronically into an ODK Central database via android platforms using ODK software (ODK Collect) and paper-based forms. Electronic data capture is prioritised where sensitive data is being collected as this data will be directly uploaded to the ODK server where it is encrypted and secured. In these cases, paper copies are to be used only as a backup where hard copies are uploaded electronically at a later date or where paper logs are required due to quality assurance compliance.

#### Security and storage

Data entered into ODK will be directly uploaded to the ODK Central server hosted via the London School of Hygiene & Tropical Medicine (LSHTM), where it will be securely stored on encrypted servers with restricted access. Daily backups will be conducted through the LSHTM server and additional weekly backups will be managed and stored offline to prevent data loss. Access to ODK Central will be strictly controlled and limited to delegated members of the study team.

Physical copies of data will be stored in a secure file with restricted access and will be destroyed when no longer in use, after a period of 10 years. Material and data from this study will then be kept for 10 years. This is a legal requirement of the funder. All data will be collected, processed, stored and handled in accordance with the Data Protection Act 2018. Where an ODK form exists that is compatible with the physical copy of data, the data will be uploaded to ODK at the earliest convenience as a primary electronic copy.

#### Processes to promote data quality

Training on electronic data collection through ODK Collect will be provided to all members of the study team and data collectors. Where appropriate, supplementary text is provided within ODK to guide the data collector to use the correct data entry format. For sensitive fields, such as participant ID, there are restrictions on answers to reduce the likelihood of entry errors. Data uploaded to ODK will be downloaded and quality assessed for errors and duplicates.

### Confidentiality {27}

All participants that enter the study will be kept anonymous. Identifiable information (name and signature) will only be located on the ICFs and household participation case report form, which will be stored securely on a locked electronic database, only accessible to delegated staff members. Participants will be assigned a unique ID number, which will be used on all other study documents and during analysis. The results of this study will be made available to the sponsors of this study and in publications, but no personal identifiers will be included.

### Plans for collection, laboratory evaluation and storage of biological specimens for genetic or molecular analysis in this trial/future use {33}

The blood samples will be tested in the Molecular Research Laboratory in Kampala in Uganda and destroyed after analysis is completed, following standardised protocols. Materials collected in this study will not be analysed outside Uganda, apart from air samples which will be analysed in laboratories at Loughborough University.

## Statistical methods

### Statistical methods for primary and secondary outcomes {20a}

#### Statistical analysis of the primary endpoint

The primary outcome will be reported as the proportion of children ≤ 5 years of age infected with malaria (%) at the cluster level. The proportion of children with malaria infection will be summarised at the cluster level and the intervention level. The outcomes will be compared across treatment arms. To address potential confounding factors and account for non-independence of data within clusters, logistic regression models will be employed. These models will incorporate a random effect for each study cluster to adjust for intra-cluster correlation.

Both unadjusted and adjusted models will be presented. The primary model will be as outlined above, and an adjusted model will include additional factors to account for variation at baseline and features that are highly predictive of the primary endpoint. These will include the following: Environmental factors known to have a strong influence on malaria incidence, including the average altitude, calculated from the mean spatial coordinate for each cluster, and the average distance from the nearest freshwater body, calculated from the mean spatial coordinate for each cluster; Use of other vector control interventions; Socio-economic status of the household; Architectural features of the household, or modification made to the household, that are related to mosquito entry; and Season of data collection.

#### Statistical analysis of the secondary endpoints

For entomological outcomes, mosquito species composition will be reported as the total abundance and species-specific proportions of mosquitoes per sample per household per night. The outcomes will be compared across treatment arms. To address potential confounding factors and account for non-independence of data within clusters, logistic regression models will be employed, as described for the primary endpoint. Mosquito density will be reported as the total abundance of mosquitoes collected stratified by species. Additionally, for female *Anopheline* mosquitoes, the abundances will be broken down by blood fed/unfed, half gravid and fully gravid status. The outcomes will be compared across treatment arms. To address potential confounding factors and account for non-independence of data within clusters, a regression model will be employed with a negative-binomial log-link function to account for overdispersion typical of count data. These models will incorporate a random effect for each study cluster to adjust for intra-cluster correlation. Environmental covariates for factors, that were not controlled for with the allocation of clusters to trial arms, will be integrated into the models. These are average altitude and the distance from the nearest freshwater source per household, calculated from the mean spatial coordinate of each cluster. Comparative analyses will assess variations in mosquito species proportions across treatment arms to gauge effects of the intervention. Mosquito sporozoite rate will be reported as the proportion of mosquitoes (%) with sporozoites present per sample. The outcomes will be compared across treatment using the same linear regressions models employed for mosquito species composition. The prevalence of resistance to pyrethroids in *Anopheles* mosquitoes will be reported as the proportion of samples that are susceptible (98–100% resistance), possibly resistant (90–97% resistance) or confirmed resistant (less than 90% resistance) per sample. The outcomes will be compared across treatment arms. To address potential confounding factors and account for non-independence of data within clusters, logistic regression models will be employed, as described for the primary endpoint.

Household survey and acceptability survey data will be summarised using basic descriptive statistics. Mixed methods will be applied for the quantitative and qualitative data that have been obtained from the entire study. Data will be entered into Excel (Microsoft) and thereafter exported into a software for analysing qualitative data, such as NVivo (Lumivero) or MAXQDA (VERBI GmbH). We will employ thematic analysis on qualitative data to group sentiments and concepts as patterns collected in the data. These findings will be reported as synthesised narratives that illustrate the most primary perceptions, experiences and acceptability of the intervention, as well as to provide additional context through description of quantitative data when appropriate.

Quantification of the temporal changes in concentration of transfluthrin present in the air will be calculated as nanograms per litre of air sampled and compared for the sampling time points. The results will be presented using summary statistics, in a graphical format and the outcomes will be compared across treatment arms. To address potential confounding factors and account for non-independence of data within clusters, logistic regression models will be employed, as described for the primary endpoint.

### Interim analyses {21b}

There is no formal interim statistical analysis planned for this study, which would require unblinding.

### Methods for additional analyses (e.g. subgroup analyses) {20b}

There is no formal statistical analysis planned for subgroups for this study.

### Methods in analysis to handle protocol non-adherence and any statistical methods to handle missing data {20c}

For non-adherence to study protocols that are classified as protocol deviations standard operating procedures (SOPs) have been established to ensure consistent management of study activities, including data collection, handling and reporting of protocol deviations and violations. Training on these SOPs is provided to all study staff, emphasising the importance of adherence and timely reporting of any deviations or violations.

Protocol deviations (PD) can be reported by any staff members through either an electronic PD case report form hosted on ODK Central, which uploads directly to the trial’s secure server, or directly to the technical officer. Both sources will be moderated by the trial technical officer. Deviations will be assessed to determine their severity (major or minor) using the PD assessment form. Corrective and preventative actions will then be documented. Major violations or incidents will be reported to the IRB following review by the PIs (within 24 h), the study sponsor (within 10 working days) and the Uganda National Council for Science and Technology (UNCST) (within 7 working days).

Efforts to enhance data quality and accuracy include both proactive training of staff and reactive measures to control data entry, aiming to minimise errors such as mis-entries. The use of electronic data capture through ODK Collect on tablets and smartphones will be prioritised, where data entry fields will be specifically designed to limit errors. This will include restrictions on sensitive variables, such as household or participant ID number. Routine quality assurance checks will be performed on these critical fields, with feedback provided directly to the data collection teams for any necessary corrections. Additionally, metadata and equipment quality queries will be embedded within the forms to help trace sources of errors and facilitate corrective follow-ups when discrepancies are detected.

## Handling missing data

Different methods will be employed to handle missingness based on the extent of missing data per variable: For < 5% data missing, we will employ multiple imputation to fill in these gaps. This method involves using existing values from similar records to replace the missing ones. For ≥ 5% data missing, we will apply multiple imputation techniques. This approach creates several different plausible fill-ins for the missing data to produce comprehensive results. Variables with an amount of missing data, which appears to be missing at random or completely at random, may be excluded from the models to ensure the integrity and reliability of our analyses.

### Plans to give access to the full protocol, participant-level data and statistical code {31c}

The statistical analysis plan and analytic code will be made open access. The data will be made available 12 months following completion of data analysis and will publicly accessible.

## Oversight and monitoring

### Composition of the coordinating centre and trial steering committee {5d}

This study is funded by an Innovate UK grant awarded to Africa Power and Arctech Innovation (Project Title: Trials of Solar-Powered, Active, Spatial Mosquito Control Dispensers to Reduce Malaria, Application Number: 10053619). Africa Power serves as the study sponsor and study administrators. The coordinating personnel at Africa Power include Managing Director, Chief Operations Officer, Financial Officer, Chief Engineer, East Africa Projects Manager and Country Director. Arctech Innovation serve as the lead organisation responsible for overall study management, clinical oversight and study monitoring. The coordinating personnel at Arctech Innovation include Co-PI, Senior Scientific Project Manager, Data Manager and Study Monitor. Malaria Consortium are the local partners and collaborators on the trial. They are responsible for the implementation and management of the trial in Uganda. The activities conducted by Malaria Consortium include, but are not limited to, household enumeration, field team training, participant recruitment and enrolment, data collection, adverse event monitoring and support in study design and data management. The coordinating personnel at Malaria Consortium include Co-PI, Senior Scientific Research Specialist, Senior Country Technical Coordinator, Surveillance, Monitoring and Evaluation Specialist, Country Monitoring and Evaluation Manager, Study Coordinator and Senior Entomologist. The coordination centre and leads from the local partner meet weekly to maintain close oversight of the trial.

A statistician from LSHTM oversees and contributes to the writing of the statistical analysis plan. The Trial Steering Committee (TSC) is composed of three individuals from LSHTM and the University of Nevada, with expertise in epidemiology and control of malaria and design of randomised control trials. They act as an independent body that oversees the overall conduct and governance of the clinical trial.

### Composition of the data monitoring committee, its role and reporting structure {21a}

A DSMB has been set up to support the safety of participants and the integrity and credibility of the ongoing trial. This includes monitoring trial data to make timely recommendations on study conduct through trial continuation, modification or termination based on observed benefits or risks. The DSMB was implemented as a supporting feature on the basis of the following conditions of this trial: Size and length of trial, nature of the disease: life threatening, nature of the study population: paediatrics, and nature of the intervention: novel spatial repellent.

The DSMB will review data related to the safety of the trial. This might include, for instance, unblinded data referring to adverse events and protocol deviations, preliminary outcomes of primary and secondary endpoints between control and treated study arms, and ad hoc reports from the trial clinician. The DSMB will review reports and determine whether to stop the study for early evidence of intervention inferiority if there are safety concerns. All meetings will take place remotely and will be set up by the DSMB Chair. Meetings are closed to the public because discussions may address confidential participant data.

### Adverse event reporting and harms {22}

The active ingredient, transfluthrin, is approved for human use by WHO and used in numerous consumer products throughout the world and has been classified by WHO as “unlikely to present acute hazard in normal use” [25]. Direct contact with transfluthrin can cause mild eye and skin irritation; however, participants are not expected to come into direct contact with the transfluthrin-treated disc and therefore there are no AEs expected with the use of this intervention. Precautions will be taken to ensure participants are well informed of its use.

All AEs and SAEs will be monitored through both active and passive monitoring systems, through reporting by study participants or observation by the study team at each communication point. Abnormal assessments that are judged by the study clinician to be clinically significant will be recorded as AEs (or SAEs if they meet the definition). The study team members will exercise medical and scientific judgement in deciding whether an abnormal finding or assessment is clinically significant.

A SAE is any untoward medical occurrence medical occurrence that results in death, is life-threatening, results in persistent or significant disability/incapacity, requires in-patient hospitalisation or prolongation of existing hospitalisation or is a congenital anomaly/birth defect in the offspring of a study subject [26].

All AEs/SAEs will be followed until they have abated, or until a stable situation has been reached. AEs/SAEs that result in a participant’s withdrawal from the study or that are present at the end of the study will be followed up (if the participant consents to this) until a satisfactory resolution or stabilisation occurs, or until a non-study-related causality is assigned.

## Reporting procedures

SAEs will be reported to the relevant IRB, sponsor and regulatory agencies according to pre-specified reporting timelines. SAEs will be reported to the Co-PIs and sponsor within 24 h after obtaining knowledge of the events. All SAEs will be reported to the Ethics Committees, within 7 days for SAEs that result in death or are life threatening followed by a period of maximum 8 days to complete the initial preliminary report. All other SAEs will be reported within a period of maximum 15 days after the PI has first knowledge of the serious adverse events. AEs or SAEs may result in study unblinding, at the discretion of the delegated staff members.

### Frequency and plans for auditing trial conduct {23}

A site initiation visit will be conducted prior to site activation to confirm preparedness for protocol execution, satisfactory site facilities, clarify the applicable regulations and requirements of the protocol, carefully review the process of implementing the protocol at the site and conduct any necessary training prior to activating the site for enrolment. An interim monitoring visit will be conducted at 6 months after product distribution to confirm participants’ rights are being protected; the study is being conducted according to the protocol and applicable regulations, confirm accurate reporting of participant safety data and study endpoints. For-cause visits will be conducted to address any unanticipated issues that arise which require training, remediation or other situations in which the site requires assistance. For-cause visits can be mandated by Arctech Innovation or Africa Power, or can be requested by Malaria Consortium. A close-out visit will be conducted to ensure that all study data and other study documentation is complete and accurate and that all study records have been reconciled. The independent DSMB will oversee trial safety through meetings at 6 months, advising the TSC on ongoing trial conduct. The TSC will meet four times once the trial has started.

### Plans for communicating important protocol amendments to relevant parties (e.g. trial participants, ethical committees) {25}

All protocol modifications will be decided by the trial Co-PI in agreement with all study collaborators (including the Sponsor Africa Power, Arctech Innovation and Malaria Consortium). Protocol amendments and changes to study-related documents will be made by the trial manager and reported to the funder and the local regulatory authorities in Uganda—Vector Control Division Research Ethics Committee (VCD—REC), UNCST and the National Drug Authority (NDA) by the PI. If necessary, protocol modifications will be communicated to the study team and VHTs, who will feedback information to participants. The clinical trials registry will be updated by the trial manager, and the revised protocol will be stored in the Investigator Site File Any protocol deviations will be documented using Protocol Deviation Form.

### Dissemination plans {31a}

Study findings will be disseminated through joint scientific publications between the research consortium, conference attendance and a final project dissemination meeting in Uganda for all stakeholders including government leads and policy makers. Additionally, findings will be disseminated at the community level through community meetings. A submission to WHO/VCAG will also be made.

## Discussion

Uganda has made significant progress towards malaria elimination, with prevalence in children under 5 years of age decreasing from 45% in 2009 to 9% in 2019 [27]. However, despite this progress, Uganda has the world’s highest malaria incidence rate. Malaria control primarily relies on IRS of insecticide programmes, targeting epidemic areas and nationwide campaigns of ITNs. Gaps in protection still remain where *Anopheles* mosquitoes have adapted to early-evening and/or outdoor biting, which avoids the effects of ITNs and IRS. Increasing levels of insecticide resistance within mosquito populations also limits the effectiveness of current interventions. The predicted risk of malaria with climate change in Uganda show upward trends, highlighting the importance of maintaining vector control interventions [28].

Spatial repellents provide a promising tool to complement existing vector control methods to address the existing gaps in protection. They can also be supplied in various formulations to fit context-specific applications which may maintain the control tool’s natural lifespan by reducing selection pressure for insecticide resistance. The spatial repellent intervention class includes products which are designed to release volatile chemicals that disperse in air and can be placed inside or around houses [29]. Currently on the market, all spatial repellents are pyrethroid-based and have both repellent and insecticidal mode of actions. These include a range of products from expensive liquid vaporizers to more accessible, inexpensive mosquito coils. However, despite the high number of registered spatial repellent products, there is insufficient evidence of their use in public health and disease control settings to inform WHO policy recommendation. A critical path of development for spatial repellent products was established, in which the VCAG identified the need to generate epidemiological data across a range of eco-epidemiological settings, specifically in Africa, to demonstrate public health impact and inform WHO policy recommendation. Several trials have now been undertaken or are underway to address this gap, including the multi-country programme, Advancing Evidence for Global Implementation of Spatial Repellents (AEGIS), which comprises three cRCTs and one operational research study across Indonesia, Kenya, Mali, Peru, Sri Lanka and Uganda. These trials cover a range of settings and contexts, including East and West African settings, efficacy against arboviral diseases and efficacy within displaced settings [30]. Recently published results from the Kenya trial show a spatial repellent product, used alongside ITNs, to reduce first time and overall malaria infections by one third, showing the significant impact spatial repellents can have in the fight against malaria [31].

This trial aims to generate evidence that will complement the ongoing spatial repellent studies in Africa, and support decision-making by WHO to recommend spatial repellent products for public health use and integration into disease control programmes. Outputs will include scientific, regulatory and social parameters and will inform future Mossie-GO™ product development and market access strategies.

## Trial status

Date recruitment started: 05 June 2024.

Status: Protocol Version 2.0 06th February 2025.

Recruitment end: June 2025.

The trial has currently completed the baseline and 6-month data collection phase, whereby all households have been recruited, received the Mossie-GO™ and baseline and 6-month sampling is complete within the sample population. Following budget restrictions, sample size for the primary endpoint after baseline was reduced from 100 participants per cluster to 60 participants per cluster, slightly reducing power estimates. Data collection at the 12-month timepoint is due to take place in June 2025.

## Data Availability

The final trial dataset with participant information anonymised will be made open access at the end of the trial. There are no contractual agreements which will limit the access of trial data for investigators.

## Appendix

**Table 1:** Restricting factors for the selection of villages as study clusters, prior to the assignment of clusters to treatment arms

Confounding factor	Explanation
Household population size	Clusters were restricted to those with more than 109 households with children ≤ 5 years of age and no more than 245 total households. This ensured a sufficient sample size per time point while maintaining feasibility based on intervention resources
Population stability	Villages with significant seasonal population shifts, such as fishing communities, were considered separately to account for fluctuations at different time points (every six months)

**Table 2:** Factors identified as potential cluster-level confounders. Only land type and study district were controlled for during random allocation, as detailed below

Confounding factor	Explanation
District	The study area spans two districts, Jinja and Buikwe, each governed independently. Districts were included as stratification factors
Socio-economic factors	Land type (urban/rural) was used as a proxy for socio-economic variation between clusters. Additional socio-economic data will be collected in household surveys for post-hoc analysis
Environmental heterogeneity	Spatial data on altitude and proximity to large water sources was unavailable before allocation of clusters. Preliminary assessments indicated that altitude variation was evenly distributed across the study area. These factors will be included in the final statistical analysis
Malaria interventions	Nationally distributed interventions, such as ITNs and IRS, were assumed to be homogeneously available across clusters. Household surveys will collect additional data on bed net usage for further analysis

## Abbreviations

AE: Adverse event
AEGIS: Advancing Evidence for Global Implementation of Spatial Repellents
CDC: Centre of Disease Control
CRCT: Cluster randomised control trial
CV: Coefficient of variation
DSMB: Data Safety Monitoring Board
FGD: Focus group discussion
HLC: Human Landing Catches
ICF: Informed Consent Form
ID: Identification
IMVs: Interim monitoring visits
IRB: International Research Board
IRS: Insecticide residual spraying
ITNs: Insecticide-treated nets
LLIN: Long-lasting insecticidal nets
LMICs: Low-middle-income countries
LSHTM: London School of Hygiene and Tropical Medicine
NDA: National Drug Authority
ODK: Open Data Kit
PCR: Polymerase chain reaction
PD: Protocol deviation
PE: Protective efficacy
PI: Principal Investigator
PIS: Participant Information Sheet
RCT: Randomised control trial
RDT: Rapid diagnostic test
REC: Research Ethics Committee
SAE: Serious adverse event
SOP: Standard operating procedure
SSA: Sub-Saharan Africa
TSC: Trial Steering Committee
UGX: Ugandan shilling
UNCST: Uganda National Council for Science and Technology
USD: United States dollar
VCAG: Vector Control Advisory Group
VCD: Vector Control Division
VHT: Village Health Team
WHO: World Health Organization
